# Separation of Linear and Cyclic Siloxanes in Pure Silica Zeolites

**DOI:** 10.1021/acs.jpcc.5c05842

**Published:** 2025-11-20

**Authors:** Jia Yuan Chng, David S. Sholl

**Affiliations:** † School of Chemical and Biomolecular Engineering, 1372 Georgia Institute of Technology, Atlanta, Georgia 30332, United States; ‡ 6146 Oak Ridge National Laboratory, Oak Ridge, Tennessee 37830, United States

## Abstract

We present a computational assessment of pure-silica zeolites for separating linear and cyclic siloxanes. We developed a force field (FF) for pure silica zeolites, when combined with our previously developed FF for siloxanes using standard Lorentz–Berthelot combining rules, shows good agreement with dispersion-corrected density functional theory calculations. We used molecular dynamics simulations to investigate diffusion of siloxanes in pure silica zeolites and identified a pure silica zeolite with the structure code FAU that enables kinetic separation of linear and cyclic siloxanes. FAU allows the diffusion of linear siloxanes (L2–L6) while excluding cyclic siloxanes. D4 siloxane does not diffuse in any of the investigated zeolites, eliminating the potential of pure silica zeolites to achieve equilibrium-based separations of linear and cyclic siloxanes.

## Graphical abstract

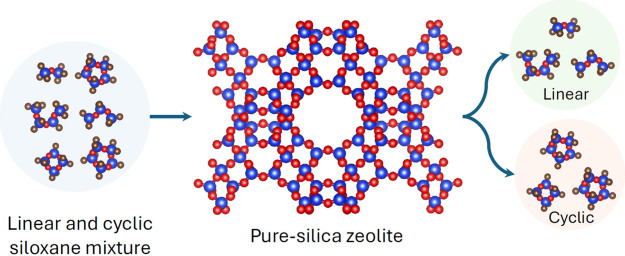

## 1 Introduction

Silicone polymers are widely used in electronics,^1^ construction,^2^ and biomedical applications^3,4^ due to their thermal and chemical stability. Cyclic siloxanes are monomers in the production of silicone polymers.^5,6^ Cyclic siloxane’s presence in the environment^7,8^ and potential health impacts have resulted in diverse regulatory frameworks, from stringent restrictions in Europe to lenient measures in other regions.^9^ The varying regulatory landscape motivates the need to remove residual cyclic siloxanes from silicone polymers.

Current approaches to remove residual cyclic siloxanes (e.g., D4, D5, and D6) from silicone polymers rely on energy-intensive processes such as fractional distillation or stripping.^10^ A recent study on the use of polymeric membranes to remove cyclic siloxanes from silicone polymers showed promising performance.^11^ However, this system may allow both cyclic and short-chain linear siloxanes to permeate. No information is currently available on the polymeric membrane’s selectivity for linear siloxanes. We assume that the membrane technology can remove residual D4, D5, and D6 from silicone polymers but also allows the permeation of L2, L3, and L4 siloxanes. This Article focuses on the separation of short-chain linear siloxanes from cyclic siloxanes using adsorption-based approaches.

Most existing studies on siloxane adsorption focus on removing specific siloxane species from biogas. Activated carbon, despite its effectiveness, suffers from limited regenerability.^12,13^ Silica gel^14,15^ and metal–organic frameworks^16–19^ (MOFs) have demonstrated good regenerability at high temperatures and deep vacuum levels, respectively. Zeolites have also been explored for siloxane adsorption. Montanari et al. examined the adsorption of D3 siloxane using NaX zeolite and found that polymerization of D3 hindered full regeneration of the adsorbent.^20^ Cabrera-Codony et al. studied seven commercial zeolites for D4 adsorption and found that zeolites with high Lewis and Brønsted acidity promoted catalytic ring-opening of D4, leading to poor desorption and sorbent regeneration.^21,22^

These prior studies indicate that cationic zeolites are unlikely to be suitable for the separation of siloxane isomers. It is possible, however, that silica zeolites may be useful for these separations. Lin et al. used Grand Canonical Monte Carlo (GCMC) and machine learning to screen 50959 hypothetical pure-silica zeolites and discovered 230 zeolites with high adsorption energy and loading for silanols and dimethylsulfone.^23^ Wang et al. studied the adsorption of L2 at low concentrations in four zeolites and porous silica materials with different pore structures, with SBA-15 showing the highest saturation adsorption capacity due to its large pore volume and MCM-41 showing the highest adsorption rate.^24^ More recently, Vega-Santander et al. investigated variants of UTD-1 for the adsorption of silanols and successfully regenerated the adsorbents by thermal and rehydroxylation treatments.^25^

Our recent work with MOFs demonstrated that both kinetic and equilibrium mechanisms can enable the separation of linear and cyclic siloxanes.^26,27^ Kinetic separation would require selective diffusion of linear siloxanes through the pores while excluding cyclic species, whereas equilibrium separation would require both classes of molecules to diffuse through the pores of the framework. Significant gaps in knowledge exist when attempting to consider pure silica zeolites for siloxane separations. The diffusion of siloxanes in pure-silica zeolites has not been quantitatively measured experimentally or computationally. In addition, no information is currently available regarding the equilibrium adsorption of mixtures of linear and cyclic siloxanes in zeolites. The potential of zeolites to achieve similar separations has, however, been demonstrated with hydrocarbons. Experimental studies by Funke et al. showed that silicalite membranes can discriminate between linear and cyclic hexanes.^28^ ZSM-5 and MFI membranes have been shown to achieve high separation factors in the separation of linear and branched hexane mixtures.^29,30^ An equilibrium adsorption study by Zhu et al. showed that configurational entropy effect favors the adsorption of linear over branched hexanes in silicalite-1.^31^ Additional examples of separation of hydrocarbon mixtures in zeolites by configurational entropy effects are discussed by Krishna.^32^

In this paper, we aim to use molecular simulations to examine whether pure silica zeolites can be used for equilibrium or kinetic separations of siloxanes. Quantitative simulation of adsorption in zeolites requires an accurate force field (FF) for adsorbate–adsorbate and adsorbate-framework interactions. The work of Lin et al. used the built-in COMPASS FF^33,34^ in the Materials Studio software for both zeolite and silanol atoms without assessing its accuracy.^23^ COMPASS FF parameters are often incompletely reported in literature and challenging to implement in open-source molecular simulation packages such as RASPA^35^ and LAMMPS.^36^

Comparing the adsorption energies computed with an FF with adsorption energies computed with dispersion-corrected Density Functional Theory (DFT) is a useful strategy to examine the accuracy of adsorbate-framework FFs, as demonstrated in studies of CO_2_ in zeolites,^37,38^ hydrocarbons in MOFs,^39^ and our previous work on siloxanes in MOFs.^40^ This comparison has not yet been applied to siloxane adsorption in zeolites. Using this approach of comparison with dispersion-corrected DFT, we present an FF for pure silica zeolite frameworks suitable for simulating siloxane adsorption. When combined with our previously developed siloxane force field through standard mixing rules,^40^ this approach yields good agreement in binding energies calculated from PBE-D3 density functional theory. Using this validated computational model, we investigate the diffusion behaviors of various siloxane species in pure silica zeolites to assess their potential for separating cyclic and linear siloxanes.

## 2 Methods

### 2.1 Pure Silica Zeolite Structures

Pure silica zeolite structures were sourced from the IZA database.^41^ We first implemented an initial screening based on the pore-limiting diameter (PLD), excluding structures with PLD less than 6 Å. In this study, we define PLD as the largest of the reported free sphere diameters among the crystallographic direction (a, b, or c) and the largest cavity diameter (LCD) as the diameter of the largest possible included sphere as provided in the IZA database. This cutoff was based on previous findings that rigid 1D MOFs with PLD below 6 Å do not allow the diffusion of linear or cyclic siloxane species.^26^

Application of this criterion to zeolite frameworks available in the IZA database as of March 2023 and excluding interrupted frameworks (indicated by a preceding dashed line) yielded 56 structures with pore sizes potentially suitable for siloxane molecules. We further refined our selection by considering only zeolites for which synthesis routes are known for pure silica structures, identified through the presence of literature references for the corresponding pure silica structure in the IZA database. The combination of these criteria resulted in a set of 14 pure silica zeolite structures. The structural parameters for these zeolites are detailed in Table S1.

### 2.2 Computational Model

To describe the interaction between siloxanes and zeolites, we applied our recently developed siloxane force field (FF) for siloxane molecules^40^ and refitted Lennard-Jones (LJ) parameters for Si and O atoms in pure silica zeolites (detailed in the following section). This siloxane FF reproduces the VLE behavior for bulk siloxanes, unlike previous force fields that have been used to model siloxanes.^40^ Dispersion interactions between the siloxanes and the zeolite atoms were described by using LJ parameters derived via Lorentz–Berthelot mixing rules. Since our siloxane FF omits point charges on the siloxane atoms, Coulombic interactions were excluded in the siloxane/zeolite interactions. The modified Hill–Sauer force field was used to account for flexibility of the zeolites.^42^ All LJ interactions were truncated at 12.8 Å with tail corrections applied. In simulations that used rigid zeolite structures, we used the crystal structure from the IZA database without further modifications. During NPT simulations with a flexible zeolite framework, the lattice constants changed by less than 0.5%. Simulation cells for zeolites were expanded to ensure that the shortest perpendicular distance was at least 26 Å, meeting the minimum image convention relative to the cutoff distance.

### 2.3 Computational Methods

Molecular dynamics (MD) simulations were performed using LAMMPS.^36^ MD simulations were run in the isothermal–isobaric (*NPT*) ensemble at *T* = 435 K and *p* = 1 atm using a Nosé-Hoover thermostat and barostat,^43^ an operating temperature and pressure consistent with prior studies on the separation of linear and cyclic siloxanes in MOFs.^26,27^ All MD simulations were carried out with a time step of 0.5 fs.

For diffusion calculations, 8 molecules were randomly inserted in each simulation supercell unless stated otherwise. Self-diffusivities were obtained from mean square displacements (MSD) that were computed using the order-*n* method.^44^ Five independent MD simulations were performed for each system. Each MD simulation comprised a 10 ns equilibration phase followed by at least 40 ns of production time to gather statistics.^45^

Adsorption isotherms for linear siloxanes were calculated using configurational bias Monte Carlo (CBMC) simulations implemented in RASPA.^35^ These simulations were performed at *T* = 435 K. Simulations consist of 100000 equilibration cycles followed by 400000 production cycles. Convergence tests to establish these simulation parameters were sufficient are detailed in our previous work.^40^

Siloxane-zeolite interaction energy calculations at the PBE-D3 density functional theory (DFT) level were performed using the Vienna ab initio simulation package (VASP).^46,47^ No atomic degrees of freedom were relaxed in these DFT calculations, and the interaction energies obtained from DFT were directly compared with the predictions of FF calculations. All single-point DFT calculations sampled the reciprocal space at the Γ point and used an energy cutoff of 400 eV.

## 3 Force Field for Siloxane-Pure Silica Zeolite Interaction

To accurately model the interactions between siloxanes and pure silica zeolites, we developed an FF that yields predictions consistent with binding energies obtained from PBE-D3 DFT calculations. Previous studies have demonstrated that force fields derived from first-principles approaches can accurately predict adsorption behavior in both zeolites^48,49^ and metal–organic frameworks (MOFs).^50,51^ To develop our force field, we first selected the six pure silica zeolites from the IZA database with the largest PLD and LCD. The structural properties of these structures are obtained directly from the IZA database^41^ and listed in Table 1.

**Table 1:** Structural Properties of the Six Selected Pure Silica Zeolites^41^

structure code	PLD (Å)	LCD (Å)
UTL	7.6	9.3
ETR	9.3	10.1
FAU	7.4	11.2
ITT	11.2	11.9
VFI	11.4	12.0
IRR	12.1	14.5

We generated 30 independent configurations of D4 siloxane in each zeolite structure using *NVT* Monte Carlo simulations at 400 K, employing the TraPPE-zeo FF^52^ for the zeolite atoms. The zeolite structures were treated as rigid in these simulations. These configurations, each containing one D4 molecule in the zeolite framework, were then used for single point energy calculations by using the PBE-D3 DFT method. Initial comparisons showed that the TraPPE-zeo FF significantly underpredicts binding energies, with a mean absolute error (MAE) of 49 kJ/mol relative to PBE-D3 calculations (open symbols in Figure 1). This discrepancy would lead to severe underpredictions of siloxane adsorption in pure silica zeolites, particularly at low loadings.

**Figure 1:**
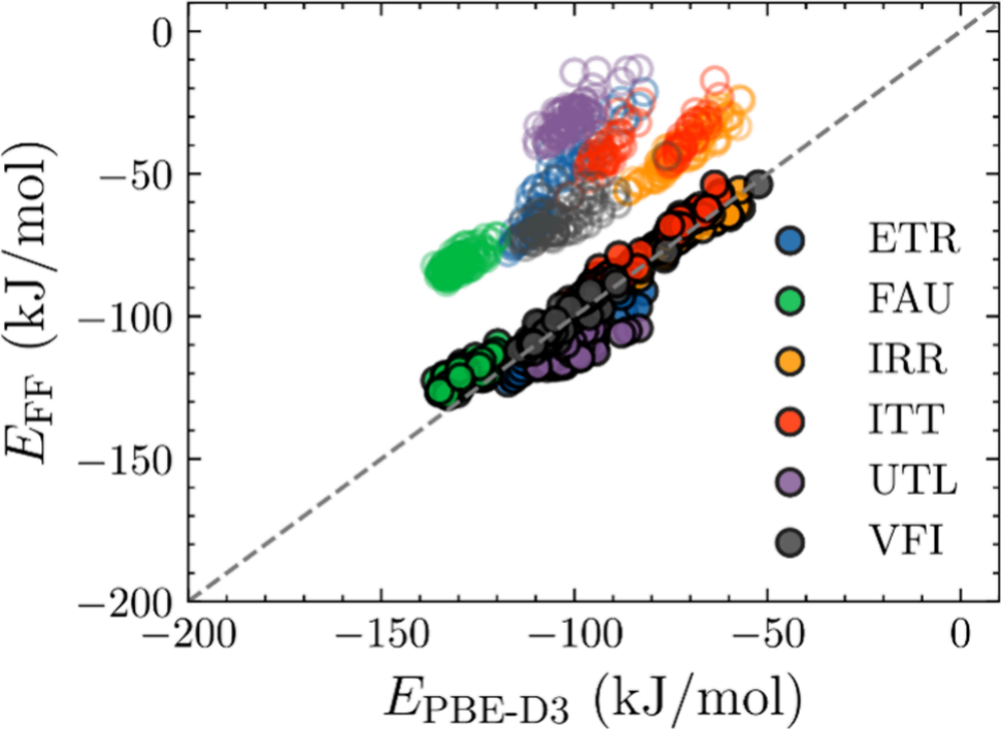
Comparison of the interaction energies of D4 in six pure-silica zeolites at the PBE-D3 level and our newly fitted FF (filled circles) and TraPPE-zeo FF^52^ (unfilled circles), with data from 30 independent configurations in each zeolite. Zeolites are represented by different colors.

To develop an improved FF, we used an iterative process to refine the Lennard-Jones parameters for Si and O atoms in pure silica zeolites. We began with initial values of σ and ε from the Universal Force Field (UFF). The adjustments focused on reducing the ε parameters for both Si and O atoms to better match the DFT-calculated binding energies, as the original UFF’s ε parameters resulted in slight overestimation. No changes were made to the original UFF’s σ parameters. The optimized LJ parameters are listed in Table 2. Figure 1 compares the binding energies of D4 in the six pure silica zeolites calculated using PBE-D3 DFT, the original TraPPE-zeo FF, and our newly fitted FF. The new FF provided binding energy predictions with a significantly lower MAE of 5 kJ/mol relative to PBE-D3 calculations, a substantial improvement over the 49 kJ/mol MAE of the TraPPE-zeo FF.

**Table 2:** Lennard-Jones Parameters for Si and O Atoms in Pure-Silica Zeolites, Fitted to Improve Agreement with PBE-D3 Binding Energies for Siloxane–Zeolite Interactions

pure-silica zeolite atoms	σ (Å)	ε (kJ/mol)
Si	3.83	1.10
O	3.12	0.21

To evaluate the transferability of our new FF, we conducted similar comparisons for linear siloxanes (L2, L3, and L4) in three randomly selected pure silica zeolites. As shown in Figure S1, the new FF demonstrates better agreement than that of TraPPE-zeo with PBE-D3 calculations across these different siloxane species. To validate the use of a Lennard-Jones only FF, we decomposed the PBE-D3 interaction energies into dispersion (D3) and nondispersion (PBE) components for the 30 configurations of L2 in FAU. As shown in Figure S2 and S3 in the SI, the dispersion term contributes the majority of the interaction energy significantly. These results show that our new FF for pure silica zeolites provides a reliable framework for modeling siloxane/zeolite interactions, offering improved accuracy over the TraPPE-zeo force field for simulating the adsorption of cyclic and linear siloxanes in pure silica zeolites.

## 4 Results and Discussion

There are no simulated or measured diffusion data of siloxanes in zeolites in the literature. Our recent work on MOFs demonstrated the feasibility of separating linear and cyclic siloxanes through both kinetic and equilibrium mechanisms.^26,27^ In that work, diffusion of siloxanes in MOFs with different pore sizes was quantitively calculated using MD simulations. Before performing computational screening of pure silica zeolites for the separation of linear and cyclic siloxanes, it is necessary to establish whether linear and cyclic siloxanes can diffuse in a representative set of pure-silica zeolites. Understanding the diffusion behavior of siloxanes would allow us to determine the feasibility of kinetic and equilibrium separation mechanisms for this application.

For our initial investigation, we selected four synthesizable pure-silica zeolites with 3D pore networks (BEC, ISV, MSE, and FAU) from Table S1. For practical application of siloxane separations based on differences in diffusion, it is desirable to focus on zeolites with 3D pore structures as they avoid complications associated with 1D materials such as single-file diffusion^53,54^ and pore blockage.^55^ We also included DON in our analysis because it has the largest PLD among all synthesizable zeolites in Table S1, making it potentially suitable for equilibrium separation if both linear and cyclic siloxanes are able to diffuse through the pores.

To efficiently identify whether these pure-silica zeolites could effectively separate linear and cyclic siloxanes, we performed a set of MD simulations to study the diffusion of siloxanes in flexible zeolite frameworks. In these simulations, we classify whether the siloxane can diffuse through the pores on time scales accessible to conventional MD simulations. If the mean squared displacement (MSD) of a siloxane species exceeded 14^2^ Å^2^ (approximately half the simulation box length) within 10 ns of simulation time, we classified it as diffusing. We first calculated the MSD of D4 siloxanes in the five zeolites to evaluate whether D4 siloxane could diffuse in each material. Since D4 did not show diffusion in any of them, all five structures were retained for subsequent evaluation of L4 diffusion to assess their potential for kinetic separation. We did not perform L4 simulations in DON since it does not allow diffusion of D4, indicating that equilibrium separation would not be feasible. Furthermore, the 1D pore structure of DON would introduce complications into kinetic separation. In zeolites that allow the diffusion of L4, we infer that the shorter chain L2 and L3 siloxanes would also diffuse. Table 3 summarizes the diffusion classifications for L4 and D4 siloxanes in the five zeolites. D4 does not show diffusion in any of the five zeolites investigated, while L4 diffuses only in FAU among the 3D zeolites, suggesting that this structure could potentially enable kinetic separations.

**Table 3:** Diffusion Classification of Siloxane Species in Five Selected Zeolites Using Flexible Framework Models, Where √ Signifies That the Siloxane Species Exhibits Diffusion As Defined in the Text and × Indicates That the Siloxane Species Does Not Display Significant Diffusion within the Material

zeolite	PLD (Å)	L4	D4
BEC	6.1	×	×
ISV	6.3	×	×
MSE	6.6	×	×
FAU	7.4	√	×
DON	8.1	–	×

The five zeolites examined above were chosen as representative candidates from Table S1 based on their variations in pore size and dimensionality. Given that the zeolite with the largest PLD in this set (DON) does not allow diffusion of cyclic siloxanes, it is unlikely that any of the synthesizable zeolites identified in Table S1 would be suitable for equilibrium separation of linear and cyclic siloxanes since this separation mechanism requires both linear and cyclic siloxanes to diffuse in the material.

To give further insight into the potential for using FAU for kinetic separations, we proceeded with additional calculations of low-loading self-diffusivity for L2, L3, L5, and L6 siloxanes in FAU, the material with 3D pores that was found to allow diffusion of linear siloxanes. The results are presented in Figure 2. In general, the self-diffusivities of linear siloxanes decrease with increasing chain length in flexible FAU. There is a significant decrease in diffusivity from L2 to L3, followed by an increase from L3 to L4, before decreasing again from L4 to L6.

**Figure 2:**
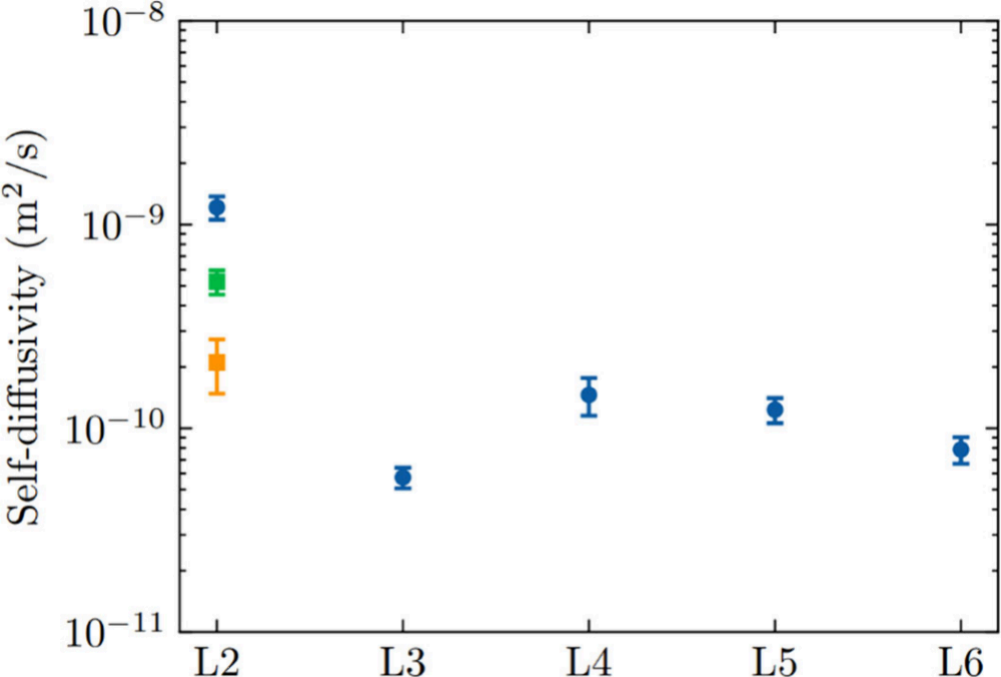
Self-diffusivity of linear siloxanes in FAU at 435 K, determined from MD simulations with a flexible zeolite structure. Blue symbols denote the diffusivity of linear siloxanes calculated at a loading of 0.3 mol/kg (49.2 mg/g). Green and orange square symbols denote the diffusivity of L2 at a loading of 77 and 98.4 mg/g respectively.

Since the MD simulations for all siloxanes in FAU were performed at a constant loading of 0.3 mol/kg, we additionally examined the loading dependence of L2′s diffusivity in FAU. The simulation described above examined L2 self-diffusion at a loading of 8 molecules/simulation cell, that is, 49.2 mg/g. At a loading of 77 mg/g, the diffusivity of L2 is 5.2 × 10^–10^ m^2^/s (green square symbol in Figure 2), and at 98.4 mg/g (comparable to the L6′s loading of 99.3 mg/g shown in Figure 2), the diffusivity further decreased to 2.1 × 10^–10^ m^2^/s (orange square symbol in Figure 2). These results confirm the expected trend that diffusivity typically decreases as loading increases, consistent with observations in loading-dependent diffusivity of hydrocarbons in porous materials.^56,57^ A nonmonotonic relationship between molecular length and diffusivity has been previously reported in simulations of linear alkanes diffusing in other nanoporous materials including silicalite,^58,59^ a zeolite with the BOF topology,^60^ and ZIF-4.^61^ The diffusivity calculations for L2 at varying loadings in FAU show that L2 maintains reasonably high diffusion rates even at higher loadings. While we have not measured the loading dependence diffusivities for L3-L6, their baseline diffusivities at 0.3 mol/kg suggest that with some expected decrease in diffusivity at higher loadings, they would still remain within the range of practical significance (10^–14^–10^–7^ m^2^/s).^45^ This shows that FAU’s capability to achieve kinetic separation would be maintained in varying conditions.

We also computed the adsorption isotherms of linear siloxanes in FAU at 435 K to characterize their adsorption behavior. The results are shown in Figure 3. Cyclic siloxanes were not included since they do not diffuse in FAU, making equilibrium separation infeasible in FAU. The isotherms show that FAU can adsorb linear siloxanes with appreciable loadings, with adsorption capacities decreasing with increasing chain length. These isotherms exhibit similar characteristics to those we predicted for MOFs in our previous work on equilibrium separation.^27^ In this previous work on MOFs, configurational entropy effects drive the preferential adsorption of linear siloxanes over cyclic siloxanes. We also observed preferential adsorption of longer linear siloxanes (L5 and L6) at lower fugacities due to stronger binding strengths relative to the shorter linear siloxanes (L2, L3, and L4), followed by a selectivity reversal at higher fugacities where entropy effects drive the preferential adsorption of shorter linear siloxanes. We can expect similar adsorption trends for linear siloxanes when the FAU is exposed to a mixture of linear and cyclic siloxanes, although the separation of linear and cyclic siloxanes in this system will be driven by kinetic effects. Overall, our investigation of siloxane diffusion in pure silica zeolites identified FAU as an adsorbent for kinetic separation of linear and cyclic siloxanes. FAU not only has the capacity to discriminate between cyclic and linear siloxanes but also facilitates the diffusion of linear siloxanes of various chain lengths. We suggest that FAU is a promising candidate for further experimental investigation of this application.

**Figure 3:**
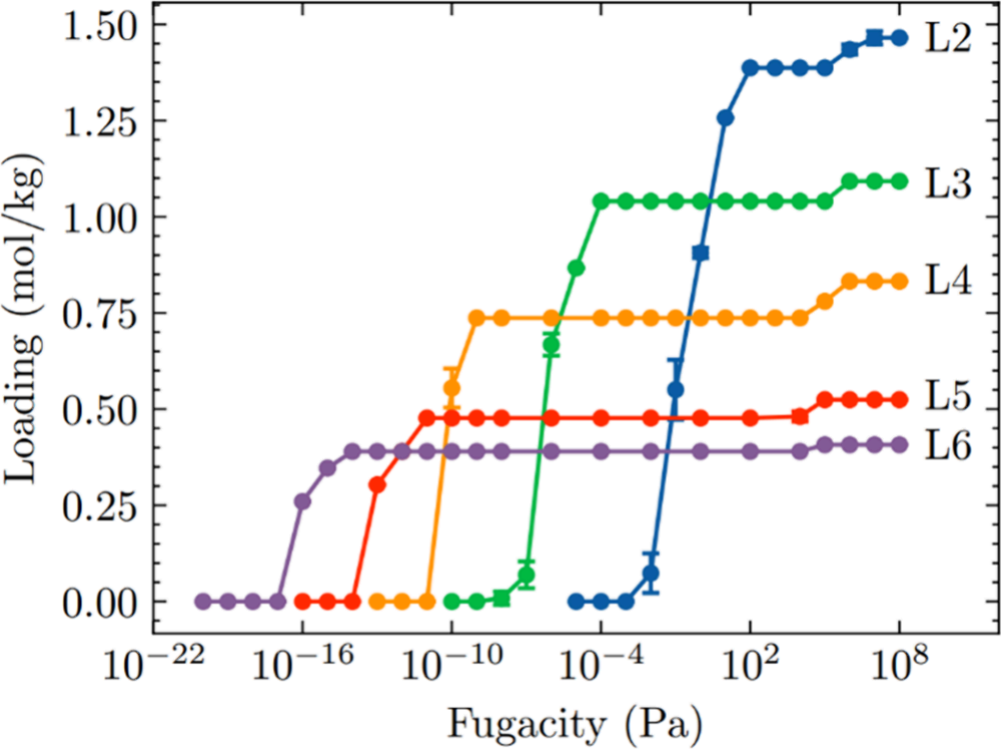
Single component adsorption isotherms of L2, L3, L4, L5, and D6 in FAU at 435 K.

## 5 Summary

We presented computational results to evaluate the potential of pure silica zeolites for the separation of linear and cyclic siloxanes. We developed a new set of Lennard-Jones parameters for pure silica zeolites that, when combined with our previously developed force field for siloxanes using standard mixing rules, yield binding energies consistent with dispersion corrected DFT calculations. Our analysis considered all synthesizable pure silica zeolites with 3D pore networks for kinetic separation. We also evaluated DON, a 1D material with the largest PLD among all synthesizable pure silica zeolites, to assess its potential for equilibrium separation. Through a series of molecular dynamics simulations, we established that D4 does not diffuse in any of the zeolites examined, eliminating the feasibility of the equilibrium-based separations of linear and branched siloxanes in these materials. Pure silica FAU is able to differentiate between cyclic and linear siloxanes while facilitating the diffusion of all linear siloxanes investigated in this study. Our results indicate that FAU is a promising pure silica zeolite for the kinetic separation of linear and cyclic siloxanes.

Recently reported pure-silica zeolites such as JZT (ZEO-3) and HZF (ZEO-5) exhibit 3D pore systems with limiting diameters of approximately 7–11 Å, comparable to or slightly larger than that of FAU. While these frameworks were deposited in the IZA Database after our data set cutoff, they represent promising candidates for future investigation, though their suitability for kinetic siloxane separation will depend on whether their enlarged pores still exclude D4 while permitting linear siloxane diffusion. FAU remains the only validated pure-silica zeolite that achieves this selectivity to date. Although our present analysis focused on pore size and diffusion behavior, future studies that examine the molecular siting of siloxanes within different zeolite frameworks could provide additional insights into how the pore geometry and topology influence adsorption and separation performance.

## Supplementary Material





## Notes

Complete collection of data for single component adsorption isotherms of linear siloxanes in FAU from CBMC simulations (XLSX)
Additional data and computational results: pore parameters for the 14 pure silica zeolites and a comparison of the interaction energies of L2, L3, and L4 in pure silica zeolites at the PBE-D3 level and FFs (PDF)
