# Supplementary material for: Separation of Linear and Cyclic Siloxanes in Pure Silica Zeolites
Source: J Phys Chem C Nanomater Interfaces. 2025 Nov 20;129(48):21463–9. doi: 10.1021/acs.jpcc.5c05842 (PMC12683633; doi:10.1021/acs.jpcc.5c05842)
Supplement: Supplementary file 2 [file jp5c05842_si_002.pdf]

Supporting Information: Separation of Linear and Cyclic Siloxanes in Pure Silica Zeolites

Jia Yuan Chng† and David S. Sholl*,‡

†School of Chemical & Biomolecular Engineering, Georgia Institute of Technology, Atlanta, GA 30332, USA

‡Oak Ridge National Laboratory, Oak Ridge, TN 37830, USA.

Email. shollds@ornl.gov

All simulation data are collated in a single Excel spreadsheet.

Sheet name	Description
L2_FAU	Unary isotherm of L2 in FAU at 435 K
L3_FAU	Unary isotherm of L3 in FAU at 435 K
L4_FAU	Unary isotherm of L4 in FAU at 435 K
L5_FAU	Unary isotherm of L5 in FAU at 435 K
L6_FAU	Unary isotherm of L6 in FAU at 435 K
SelfDiffusivity	Self-diffusivities of linear siloxanes in FAU calculated from MD simulations
DFT_FF_compare	PBE-D3 DFT, new FF and TraPPE-zeo data for siloxane-zeolite binding energies

Table S1: Structural parameters of 14 synthesizable pure silica zeolites with pore limiting diameter (PLD) greater than 6 Å obtained from the IZA database.[1]

Structure code	PLD (Å)	LCD (Å)	Pore channel dimension
BEC	6.1	7.1	3D
SFE	6.3	6.7	1D
ISV	6.3	7.0	3D
IFR	6.4	7.2	1D
MOR	6.5	6.7	1D
MSE	6.5	7.1	3D
OFF	6.5	7.0	1D
ATS	6.8	7.3	1D
MEI	6.9	8.1	1D
CFI	7.3	7.5	1D
FAU	7.4	11.2	3D
AFI	7.4	8.3	1D
MAZ	7.5	8.1	1D
DON	8.1	8.8	1D

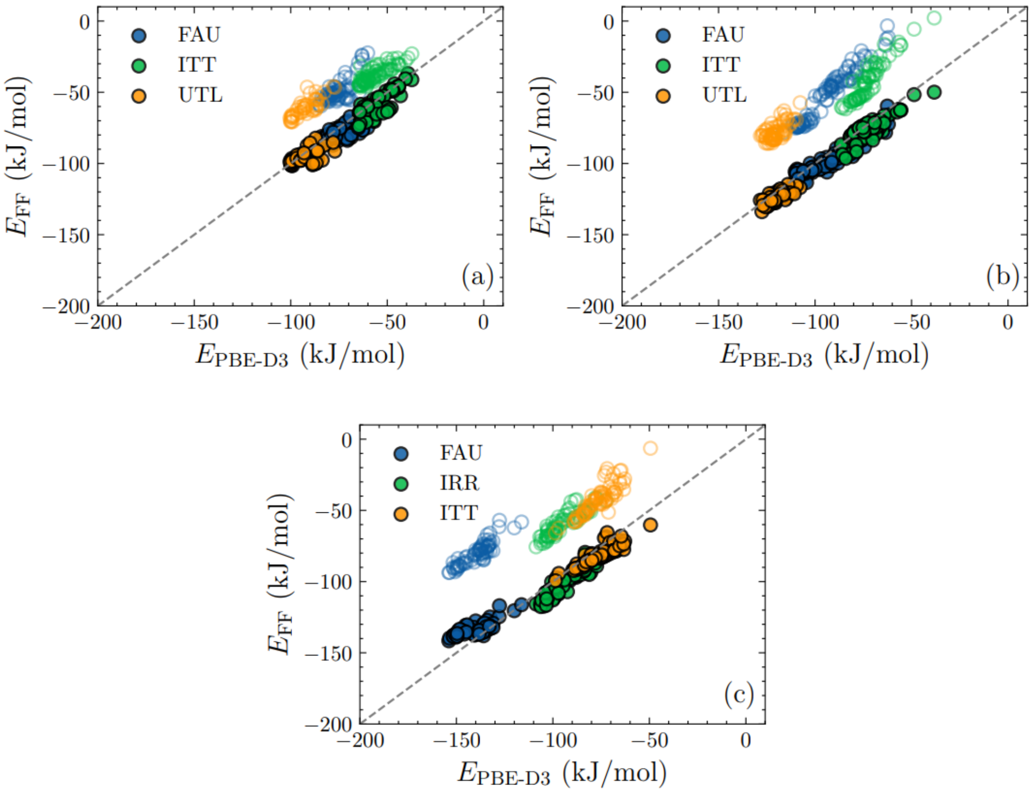

Figure S1: Comparison of the interaction energies of (a) L2, (b) L3 and (c) L4 in 3 randomly selected pure-silica zeolites at the PBE-D3 level and our new FF (filled circles) and TraPPE-zeo[2] FF2 (unfilled circles), with data from 30 independent configurations in each pure-silica zeolite. Mean absolute error (MAE) of the binding energies are tabulated in Table S2.

Table S2: Mean absolute error (MAE) of the binding energies of L2, L3 and L4 in 3 randomly selected pure-silica zeolites at the PBE-D3 level and our new FF and TraPPE-zeo FF.[2]

Molecule	New FF (kJ/mol)	TraPPE-zeo FF (kJ/mol)
L2	4.6	25
L3	4.6	38
L4	5.3	43

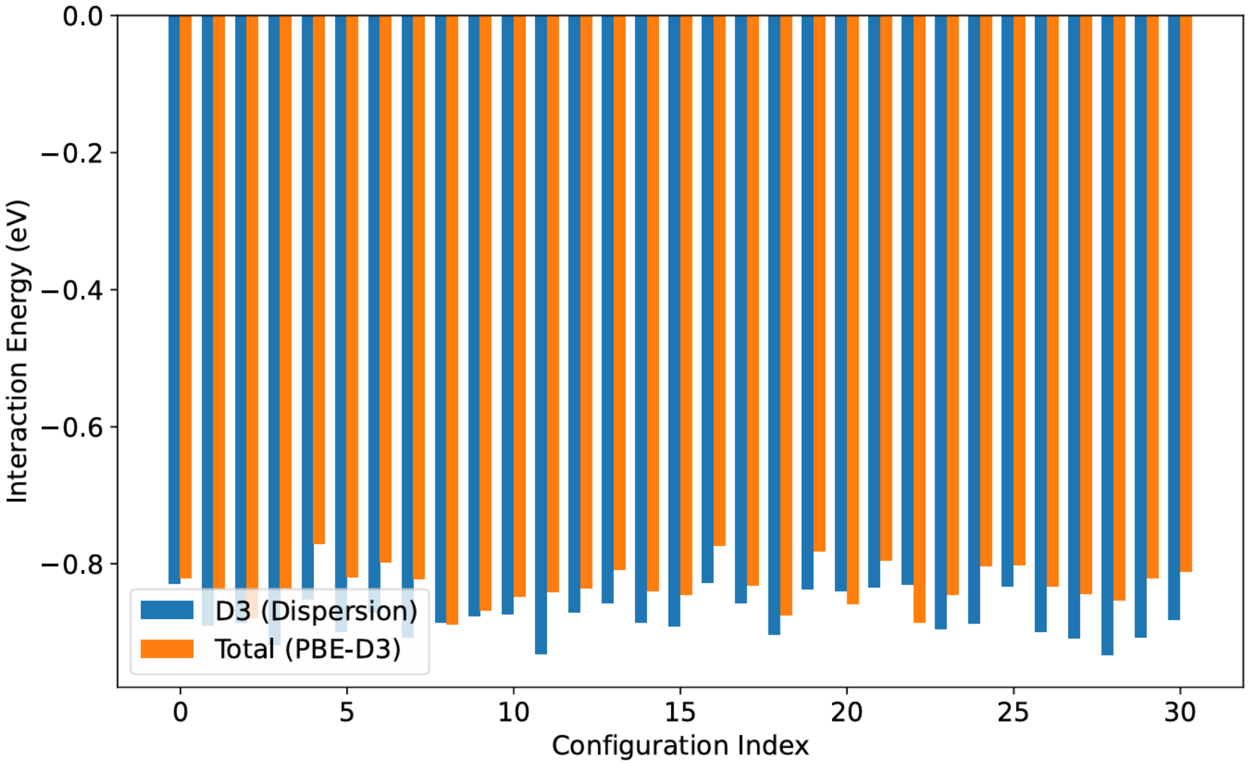

Figure S2: Interaction energies of L2 in pure-silica FAU across 30 independent configurations, decomposed into total PBE-D3 energy (orange) and D3 (blue) component.

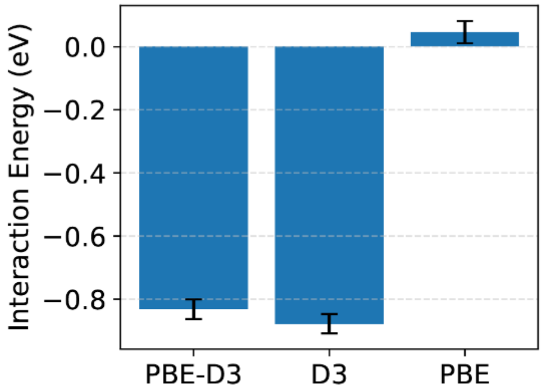

Figure S3: Bar chart comparing the average PBE-D3 interaction energy, D3 (dispersion-only) and PBE (non-dispersion) component for L2 siloxane in FAU across 30 configurations.

References

1. Baerlocher, C.; McCusker, L. B. IZA Structure Database. http://www.iza-structure.org/databases (accessed March 1, 2023).
2. Bai, P.; Tsapatsis, M.; Siepmann, J. I. TraPPE-Zeo: Transferable Potentials for Phase Equilibria Force Field for All-Silica Zeolites. The Journal of Physical Chemistry C 2013, 117, 24375–24387.
